# Improved Passive State Preparation–Continuous Variable Quantum Key Distribution Scheme Based on Non-Gaussian Operations

**DOI:** 10.3390/e28050497

**Published:** 2026-04-27

**Authors:** Hao Luo, Yijun Wang, Hang Zhang, Jiajia Zhong

**Affiliations:** 1 School of Automation, Central South University, Changsha 410083, China; 194601043@csu.edu.cn (H.L.); xxywyj@sina.com (Y.W.); 2 School of Information Engineering, Gannan University of Science and Technology, Ganzhou 341000, China; 3 State Grid Jiangxi Ganzhou Power Supply Company, Ganzhou 341000, China; jiajia0823@hotmail.com

**Keywords:** continuous variable quantum key distribution, passive state preparation, non-Gaussian operations, secret key rate

## Abstract

Passive state preparation–continuous variable quantum key distribution (PSP-CVQKD) protocol inherits the advantage of high secret key rate (SKR) of CVQKD, while overcoming the drawback of complex modulation equipment in GMCS-CVQKD. In recent years, it has received extensive attention in the experimental field. Even so, short transmission distance remains its prominent issue. In this paper, a scheme for introducing non-Gaussian operations into PSP-CVQKD in optical fiber links is proposed. We derive the input–output relationship of the system, as well as the calculation formulas for the success probability and SKR, when non-Gaussian operations are introduced at both sides of the channels respectively. The results indicate that the improved PSP-CVQKD scheme is feasible in enhancing the SKR performance and can effectively increase the transmission distance. Our scheme provides beneficial ideas for further in-depth research on non-Gaussian operations and the performance improvement of other PSP-CVQKD protocols.

## 1. Introduction

Quantum key distribution (QKD) is a technology based on the fundamental principles of quantum mechanics [1,2]. Compared with quantum teleportation (QT) and quantum dense coding (QDC), QKD has been the most in-depth researched field, due to its relatively low reliance on quantum entanglement and better integration with classical communication technologies. As one of the two technical routes of QKD, CVQKD has advantages over its counterpart, DVQKD, such as simple preparation, low detection cost and high SKR, which has enabled CVQKD to receive the most extensive research and even initiated the commercialization process in specific scenarios [3,4,5,6,7,8,9]. However, a critical issue hindering its commercialization is transmission distance.

Recent studies show that the problems above can be effectively improved in entanglement-based CVQKD (EB-CVQKD) by using non-Gaussian operations. EB-CVQKD [10,11] achieves key distribution by utilizing highly pure quantum entangled states. Compared with coherent-state CVQKD, EB-CVQKD demonstrates a stronger resistance to excess noise [11], hereby enabling longer transmission distance and higher SKR. One effective approach of improving the performance of EB-CVQKD is to increase the degree of entanglement; extensive research has shown that Gaussian operations are ineffective in this regard [12,13,14]. In contrast, entanglement states distillation can be effectively achieved through non-Gaussian operations [15,16,17,18,19,20,21,22]. Photon addition, photon subtraction and photon catalysis are common non-Gaussian operations. The basic idea is to add or subtract photons in one or two modes of EB-CVQKD, or catalyze the original optical signal, ultimately improving the SKR and transmission distance.

For instance, researchers have applied the photon subtraction to the measurement-device-independent CVQKD (MDI-CVQKD) protocol, effectively extending the transmission distance and key rate [23]. In 2019, the novel non-Gaussian operations of photon catalysis were successfully applied to CVQKD [24], further improving system performance. Introducing zero-photon catalysis in discrete modulation (DM) MDI-CVQKD can also achieve higher SKR, reasonably increase the optimal variance, and in some cases, extend the transmission distance [25]. Performing photon addition operations on the left side of the entanglement source has higher success probability and SKR and longer transmission distance than performing the same operations on the right side [26]. Even when the entanglement source is located in the untrusted part, EB-CVQKD based on photon subtraction can still offer better performance in resisting attacks and extending the secure transmission distance [15]. In summary, numerous studies have shown [27,28,29,30,31,32,33] that non-Gaussian operations can effectively enhance system performance in many CVQKD schemes.

However, whether the introduction of non-Gaussian operations in PSP-CVQKD can improve system performance has not been fully studied yet. As a relatively new member of the CVQKD protocol series, PSP-CVQKD does not require active Gaussian modulation, effectively reducing system complexity and improving system stability.

From the perspective of the eavesdropper Eve, it is impossible to distinguish whether the stolen quantum state is from PSP-CVQKD, GMCS-CVQKD, or EB-CVQKD. In other words, the three CVQKD protocols are equivalent in terms of security [34]. In the PSP-CVQKD scheme, Alice directly uses a thermal source to generate the required quantum state without the need for amplitude modulators and phase modulators, greatly simplifying the device volume, complexity and cost. Then she uses a beam splitter (BS) to split the light into two parts, estimates the transmitted part by measuring the other one. This estimated value is then processed with Bob’s measurement value to extract the secret key.

This paper presents an improved PSP-CVQKD scheme based on non-Gaussian operations; we study the success probability of non-Gaussian operations and the input–output relationship of the system and analyze and compare the performance such as transmission distance and SKR when different non-Gaussian operations are respectively applied at both sides of the system and when no non-Gaussian operations are applied.

The paper is organized as follows. Section 2 introduces the main device in non-Gaussian operations, as well as the principles and types of non-Gaussian operations. Section 3 introduces the classical PSP-CVQKD protocol without non-Gaussian operations, and derives the input–output relationship and key rate calculation method. Section 4 elaborates on the principle and working process of our proposed scheme and derives the calculation methods for the input–output characteristic functions (CF), the characteristic matrix (CM), the SKR, and the success probability of the non-Gaussian operations. In Section 5 and Section 6, we analyze the system performance when non-Gaussian operations are applied to Alice’s and Bob’s sides respectively. The conclusion of the paper is presented in Section 7.

## 2. PSP-CVQKD Protocol

**Figure 1:**
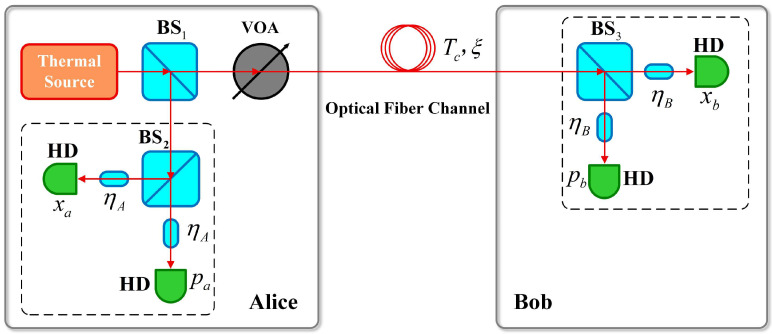
Schematic diagram of the PSP-CVQKD protocol.

In order to achieve the research target of this paper, first of all, the characteristics of the PSP-CVQKD protocol should be studied. Figure 1 shows the schematic diagram of the PSP-CVQKD protocol.

First, the thermal source outputs the optical signal, which is split into two equal parts by $BS_{1}$; among them, mode a is sent to Alice’s own heterodyne detector with an efficiency of $\eta_{A}$, and the measurement results are $x_{a}$ and $p_{a}$. After passing through a variable optical attenuator (VOA), mode b is transmitted to Bob via an untrusted optical fiber channel (with the transmissivity $T_{c}$ and excess noise ξ). After being detected by a heterodyne detector with efficiency of $\eta_{B}$, the measurement results $x_{b}$ and $p_{b}$ are obtained. By repeating the above process multiple times, both parties accumulate sufficient raw keys; then, the post-processing process can be proceeded to obtain the final keys.

The input–output relationship and the CF of the input–output of PSP-CVQKD can be expressed using the density operator as [35]

(1) $$\chi_{out}(\beta )=\exp[-\frac{2\bar{n}+1}{2}(1-T_{c}){\left|\beta \right|}^{2}]\chi_{in}(\sqrt{T_{c}}\beta ).$$

Here, $\chi_{in}$ and $\chi_{out}$ represent the CFs of the input and output states, respectively, where $T_{c}$ is the channel transmissivity and $\bar{n}$ represents the average number of input photons, $\bar{n}=\xi T_{c}/2(1-T_{c})$. Based on Equation (1), after mode b passes through the optical fiber channel, the output CF can be derived as

(2) $$\chi_{out}(\alpha ,\beta )=\exp[-\frac{2\bar{n}+1}{2}(1-T_{c}){\left|\beta \right|}^{2}]\chi_{in}(\alpha ,\sqrt{T_{c}}\beta ).$$

Here $\alpha =q_{1}+ip_{1}$ and $\beta =q_{2}+ip_{2}$. Due to the equivalence of the PSP-CVQKD and EB-CVQKD, the CF expression of any mode in PSP-CVQKD can be written as

(3) $$\chi_{in}(\alpha ,\beta )=\exp[-\frac{V}{2}({\left|\alpha \right|}^{2}+{\left|\beta \right|}^{2})+\frac{\sqrt{V^{2}-1}}{2}(\alpha \beta +\alpha^{*}\beta^{*})].$$

Here, $V=(1+\lambda^{2})/(1-\lambda^{2})$. Substituting Equation (3) back into Equation (2), the input–output relationship and the CM of the system after transmission through the optical fiber channel in the PSP-CVQKD protocol are respectively expressed as follows:

(4) $$\chi_{out}(\alpha ,\beta )=\exp[(-a_{1}{\left|\alpha \right|}^{2}+a_{2}{\left|\beta \right|}^{2})/2+a_{3}(\alpha \beta +\alpha^{*}\beta^{*})/2],$$

(5) $$V_{AB}=\left(\begin{array}{cc} a_{1}I & a_{3}\sigma_{z}\\ a_{3}\sigma_{z} & a_{2}I \end{array}\right),$$

where $a_{1}=V$, $a_{2}=T_{c}(V+\chi_{\xi })$, $\chi_{\xi }=(1-T_{c})/T_{c}+\xi$, $a_{3}=\sqrt{T_{c}(V_{c}^{2}-1)}$, $I=\left(\begin{array}{cc} 1 & 0\\ 0 & 1 \end{array}\right)$, $\sigma_{z}=\left(\begin{array}{cc} 1 & 0\\ 0 & -1 \end{array}\right)$, and $\chi_{\xi }$ represents the total excess noise in the channel.

SKR: Since it is equivalent to GMCS-CVQKD in terms of security, considering the conditions of collective attacks and Bob’s reverse reconciliation, the SKR of PSP-CVQKD in the asymptotic limit can be calculated using the following formula [36,37]:

(6) $$K_{PSP}=K_{GMCS}=\beta_{RECO}I_{AB}-\chi_{BE}.$$

$\beta_{RECO}$ represents the reconciliation efficiency of Bob, $I_{AB}$ is the Shannon mutual information between Alice and Bob, and $\chi_{BE}$ indicates the upper limit of the information that Eve can obtain from Bob, known as the Holevo bound.

In fact, since thermal state is more likely to exhibit broadband spectrum characteristics in practical applications, the analysis method for the continuous mode is more accurate when conducting security analysis. This paper only focuses on the ideal single mode situation, and the complex continuous mode will be studied in future research.

## 3. Non-Gaussian Operations

**Figure 2:**
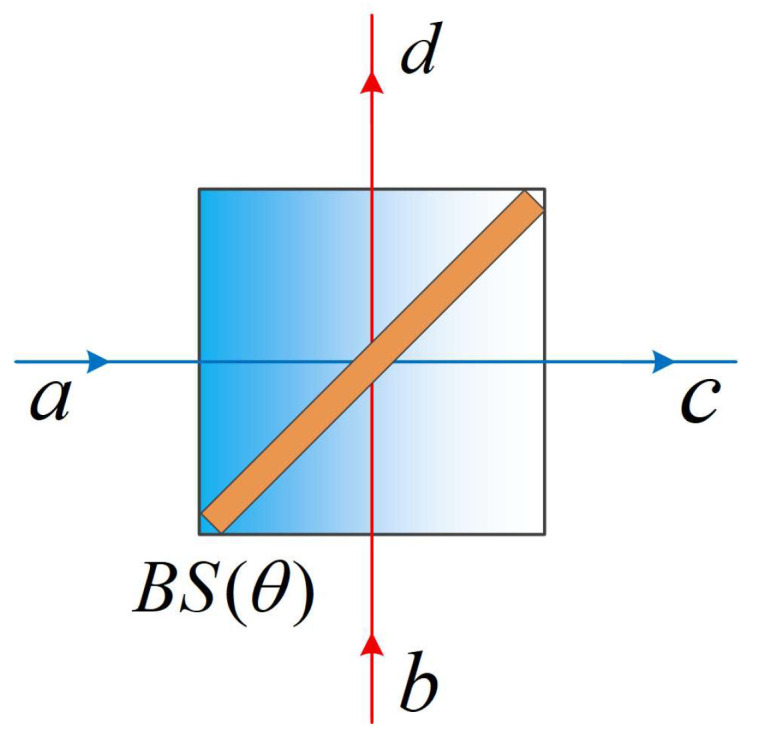
Quantum model of beam splitter.

In quantum optics, non-Gaussian operations can transform Gaussian quantum states into non-Gaussian quantum states, which plays a significant role in both theoretical research and practical applications. The BS is widely used to implement non-Gaussian operations. The transmissivity T and the reflectance R=1−T are key parameters, and its quantum model is shown in Figure 2.

When performing non-Gaussian operations using a BS, suppose the input states are $|\psi_{in}\rangle$ and the Fock state is |m〉, respectively. A conditional measurement of the Fock state |n〉 is performed at the output port corresponding to |m〉, the port corresponding to $|\psi_{in}\rangle$ outputs $|\psi_{out}\rangle$, and the relationship between the input and output states can be described as follows:

(7) $$|\psi_{out}\rangle =N_{m,n\;a}{\langle n|B(\theta )|m\rangle }_{a}|\psi_{in}\rangle .$$

Here, B(θ) is the BS operator and $N_{m,n}$ is the normalization factor. By using the technique of integration within an ordered product (IWOP) [38,39] and the representation of Fock states in the coherent state, expressions of the matrix elements $B_{n,m}\equiv \langle n|B(\theta )|m\rangle$ corresponding to three common non-Gaussian operations can thus be derived [40].

Photon Addition: n=0, where photons are added to the signal channel, and

(8) $$B_{0,m}=\langle 0|B(\theta )|m\rangle =\frac{{\left(-\sin\theta \right)}^{m}}{\sqrt{m!}}b^{†m}e^{b^{†}b\ln\cos\theta }.$$

Photon Subtraction: m=0, photons are subtracted from the signal channel, and

(9) $$B_{n,0}=\langle n|B(\theta )|0\rangle =\frac{\tan^{n}\theta }{\sqrt{n!}}b^{n}e^{b^{†}b\ln\cos\theta }.$$

Photon Catalysis: m=n, neither photons are subtracted nor added, and

(10) $$B_{m,m}=\langle m|B(\theta )|m\rangle =\cos^{m}\theta :L_{m}(b^{†}b\tan^{2}\theta ):e^{b^{†}b\ln\cos\theta }.$$

**Figure 3:**
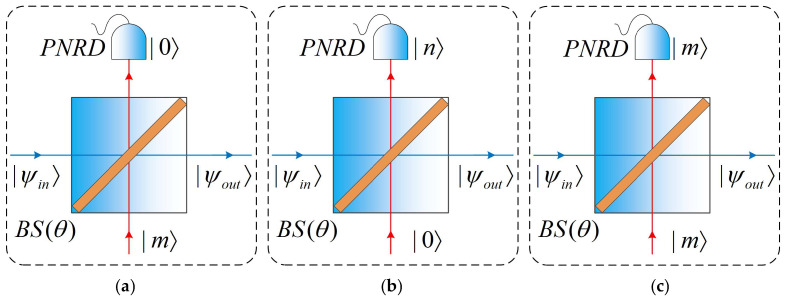
Schematic diagram of common non-Gaussian operations implemented using BS: (**a**) photon addition; (**b**) photon subtraction; (**c**) photon catalysis.

Figure 3a, Figure 3b, and Figure 3c respectively represent photon addition, photon subtraction, and photon catalysis, and these operations all employ ideal BS and conditional measurement. In the conditional measurement method, photon counting is achieved using a photon-number-resolving detector (PNRD) that operates based on the principle of time division multiplexing (TDM) [41,42].

Success Probability: It should be noted that non-Gaussian operations are non-deterministic; that is, the successful implementation of non-Gaussian operations occurs with a certain probability. Therefore, deriving the expression for the success probability plays a crucial role in implementing non-Gaussian operations and analyzing system performance.

Using the definition formula of the non-Gaussian term $G_{m,n}(\alpha ,\beta )$,

(11) $$G_{m,n}(\alpha ,\beta )=\frac{(1-\lambda^{2})\lambda^{2n}{\left(-R\right)}^{m+n}}{{\left(1-{\bar{\lambda }}^{2}\right)}^{m+n+1}m!n!}\times \frac{\partial^{2(m+n)}}{\partial t_{1}^{m}\partial t_{2}^{m}\partial t_{3}^{n}\partial t_{4}^{n}}e^{-t_{1}t_{2}+B_{2}t_{1}+B_{2}^{*}t_{2}}\times e^{-t_{3}t_{4}+B_{1}^{*}t_{3}+B_{1}t_{4}-B_{3}(t_{1}t_{4}+t_{2}t_{3})}{|}_{t_{1},t_{2},t_{3},t_{4}=0}.$$

Here $B_{1}=\frac{\alpha -\bar{\lambda }\beta^{*}}{\sqrt{1-{\bar{\lambda }}^{2}}}$, $B_{2}=\frac{\beta -\bar{\lambda }\alpha^{*}}{\sqrt{1-{\bar{\lambda }}^{2}}}$, and $B_{3}=\sqrt{T}\frac{1-\lambda^{2}}{\lambda R}$, and the success probabilities of photon addition (n=0), photon subtraction (m=0), and photon catalysis (m=n) can be derived as follows [43]:

(12) $$G_{m,0}(0,0)=\frac{(1-\lambda^{2})R^{m}}{{\left(1-{\bar{\lambda }}^{2}\right)}^{m+1}},$$

(13) $$G_{0,n}(0,0)=\frac{(1-\lambda^{2})\lambda^{2n}}{{\left(1-{\bar{\lambda }}^{2}\right)}^{n+1}}R^{n},$$

(14) $$G_{m,m}(0,0)=D_{m}\frac{(1-\lambda^{2})T^{m}/{\left(m!\right)}^{2}}{W(t,\tau )-{\bar{\lambda }}^{2}W(t/T,\tau /T)}.$$

Here, $D_{m}=\frac{\partial^{2m}}{\partial t^{m}\partial \tau^{m}}{\left\{\cdot \right\}}_{t,\tau =0}$ and W(x,y)=(1−x)(1−y). $G_{m,n}(0,0)$ refers to the success probability when m photons are injected into one port and n photons are detected at the corresponding output port. Specifically, when single-photon catalysis (m=n=1) is implemented, the success probability satisfies

(15) $$G_{1,1}(0,0)=\frac{\left(1-\lambda^{2}\right)}{{\left(1-\lambda^{2}T\right)}^{3}}[\lambda^{2}R^{2}+T{\left(1-\lambda^{2}\right)}^{2}].$$

By using Equations (12)–(15) above, the relationship between the success probability, the transmissivity T and the squeezing parameter λ can be derived when different non-Gaussian operations are implemented.

## 4. PSP-CVQKD Protocol with Non-Gaussian Operations

The input–output relationship: The Weyl expansion of the density operator is known to be

(16) $$\rho =\int_{-\infty }^{\infty }\frac{1}{\pi }d^{2}\gamma \chi (\gamma )D(-\gamma ).$$

Here χ(γ) is the CF of the operator, and $D(-\gamma )=e^{\left(\gamma a^{†}-\gamma^{*}a\right)}$ is the translation operator. For an ideal BS, if $\rho_{in,b}$ is the arbitrary state input from the b port, then the corresponding output state is

(17) $$\rho_{out,b}=\mathrm{Tr}{[B(\theta )\rho_{in,b}|m\rangle }_{b'b'}{\langle m|B^{†}|n\rangle }_{b'b'}\langle n|],$$

where |m〉 is the auxiliary state input from the b' port, and using Equation (16), the input–output relationship of the CF for any input state is derived as

(18) $$\chi_{out}(\beta )=\int_{-\infty }^{\infty }\frac{1}{\pi R}d^{2}\gamma \chi_{in}(\gamma )\chi_{m}(\gamma_{1})\chi_{n}(\gamma_{2}).$$

Here $\gamma_{1}=\beta /\sqrt{R}-\gamma \sqrt{T/R}$ and $\gamma_{2}=\gamma /\sqrt{R}-\beta \sqrt{T/R}$, where $\chi_{in}$, $\chi_{m}$ ($\chi_{n}$) are the CFs of the input state and the Fock state, respectively, and $\chi_{m}(\gamma_{1})=\exp(-{\left|\gamma_{1}\right|}^{2}/2)L_{m}({\left|\gamma_{1}\right|}^{2})$ and

(19) $$L_{m}(x)=\frac{e^{x}}{n!}\frac{d^{n}}{dx^{n}}(e^{-x}x^{n}).$$

Here $L_{m}(x)$ is the standard Laguerre polynomial, which has the following relationship with the bivariate Hermite polynomial: $H_{m,m}(x,y)$

(20) $$L_{m}(xy)={\left(-1\right)}^{m}H_{m,m}(x,y)/m!.$$

When any mode in PSP-CVQKD inputs to the ideal BS, the corresponding output can be calculated using Equation (18):

(21) $$\chi_{out}(\alpha ,\beta )=\chi_{in,\bar{\lambda }}(\alpha ,\beta )G_{m,n}(\alpha ,\beta ).$$

$\chi_{in,\bar{\lambda }}(\alpha ,\beta )$ is the CF of the passive state preparation and $\bar{\lambda }=\lambda \sqrt{T}$. Further, the normalized CF expression can be derived as

(22) $$\chi {'}_{out}(\alpha ,\beta )=\frac{\chi_{in,\bar{\lambda }}(\alpha ,\beta )G_{m,n}(\alpha ,\beta )}{G_{m,n}(0,0)}.$$

where $G_{m,n}(0,0)$ is the success probability of the non-Gaussian operations.

## 5. PSP-CVQKD Scheme Based on Non-Gaussian Operations Implemented at Alice’s Side

**Figure 4:**
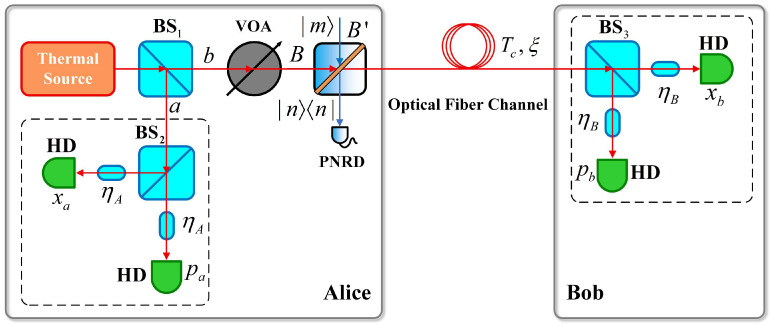
PSP-CVQKD scheme based on non-Gaussian operations implemented at Alice’s side.

Next, we discuss the case where non-Gaussian operations are implemented at Alice’s side, as shown in Figure 4. The quantum state generated by Alice is input from the B port of the ideal BS, the auxiliary Fock state |m〉 is input from B', and the conditional measurements are performed at the corresponding output ports.

Suppose that mode a is used for Alice’s heterodyne detection, then mode b is transmitted to Bob. We can derive the output CF of mode b by substituting Equation (3) into Equation (22). Then, we can substitute this CF into Equation (2) and the output CF at Bob’s side can be obtained:

(23) $$\chi_{out}(\alpha ,\beta )=\frac{\exp(-\frac{\xi T_{c}+R_{c}}{2}{\left|\beta \right|}^{2})}{G_{m,n}(0,0)}\chi_{in,\bar{\lambda }}(\alpha ,\sqrt{T_{c}}\beta )\times G_{m,n}(\alpha ,\sqrt{T_{c}}\beta ),$$

**Figure 5:**
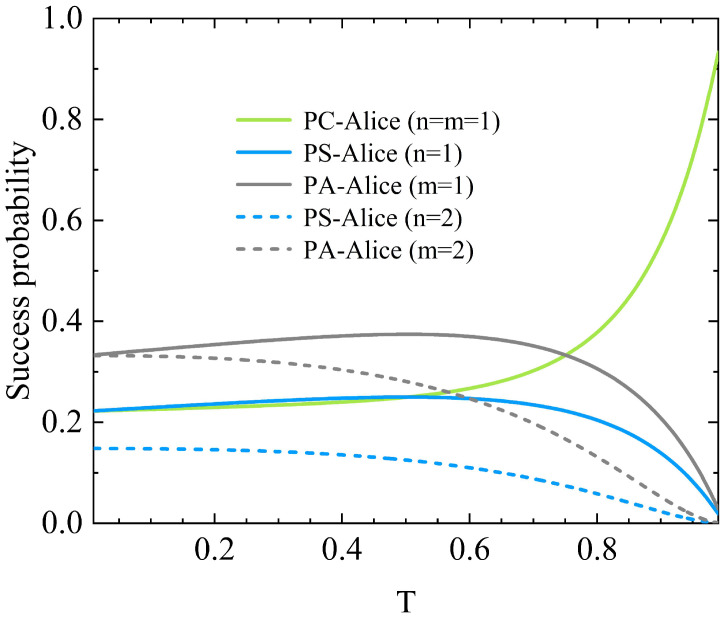
Success probabilities of non-Gaussian operations as a function of T.

Using Equations (12)–(15), we studied the variation in the success probability with T when three non-Gaussian operations were respectively implemented at Alice’s side, shown in Figure 5, where PS‐Alice(n=m=1), PS‐Alice(n=1) and PA‐Alice(m=1) represent single-photon catalysis, single-photon subtraction and single-photon addition, respectively. PS‐Alice(n=2) and PA‐Alice(m=2) respectively represent photon subtraction and photon addition when n=2 and m=2.

It can be seen from Figure 5 that only the success probability of single-photon catalysis continuously increases with the increase in T. Especially in the interval where T>0.6, it rises rapidly and approaches nearly 100% when T=1. When the photon addition operation is implemented, starting from the low transmissivity range, the success probability of single-photon addition is higher than that of m=2, and this advantage becomes more obvious as T increases. When T≈0.5, the success probability approaches 40%, and then it drops rapidly. The success probability of single-photon subtraction is consistently higher than that of n=2 throughout. In the low transmissivity range, the success probability of single-photon subtraction is almost the same as that of single-photon catalysis, reaching a maximum success probability of about 25% when T≈0.5. Similarly, in the high transmissivity range, the success probability of single-photon subtraction drops sharply. Overall, the success probability of photon addition for m=1 and m=2 is always higher than that of photon subtraction for the corresponding n=1 and n=2. In the medium and low transmissivity range, the success probability of single-photon catalysis is significantly lower than that of photon addition, but in the high transmissivity range, the situation is exactly the opposite.

Next, we discuss the secret key performance of the system; the lower bound of the key rate $K_{ANGO}$ is given by

(24) $$K_{ANGO}=P_{NGO}(\beta_{RECO}I_{AB}-\chi_{BE}).$$

Here $P_{NGO}$ represents the non-Gaussian operation success probability. Using the method provided in the literature [43] and Equation (23), we can derive the expression of $V_{AB}$ as

(25) $$V_{AB}=\left(\begin{array}{cc} uI & w\sigma_{Z}\\ w\sigma_{Z} & vI \end{array}\right)=\left(\begin{array}{cc} XI & \sqrt{T_{c}}Z\sigma_{Z}\\ \sqrt{T_{c}}Z\sigma_{Z} & T_{c}(Y+\chi_{\xi })I \end{array}\right).$$

X, Y and Z have different expressions when different non-Gaussian operations are implemented. When implementing photon addition

(26) $$X_{a}=2m\;\sinh^{2}\bar{r}+\cosh2\bar{r},$$

(27) $$Y_{a}=2m\;\cosh^{2}\bar{r}+\cosh2\bar{r},$$

(28) $$Z_{a}=(m+1)\sinh2\bar{r}.$$

When implementing photon subtraction

(29) $$X_{s}=2n\;\cosh^{2}\bar{r}+\cos h2\bar{r},$$

(30) $$Y_{s}=2n\;\sinh^{2}\bar{r}+\cosh2\bar{r},$$

(31) $$Z_{s}=(n+1)\sinh2\bar{r}.$$

When implementing photon catalysis

(32) $$X_{c}=Y_{c}=D_{m}\frac{2N_{m,m}^{2}W_{0}W(t,\tau )}{{\left[W(t,\tau )-{\bar{\lambda }}^{2}W(t/T,\tau /T)\right]}^{2}}-1,$$

(33) $$Z_{c}=D_{m}\frac{2N_{m,m}^{2}W_{0}\bar{\lambda }(1-t)(1-\tau /T)}{{\left[W(t,\tau )-{\bar{\lambda }}^{2}W(t/T,\tau /T)\right]}^{2}.}$$

where $W_{0}=(1-\lambda^{2})T^{m}/{\left(m!\right)}^{2}$, and

(34) $$N_{m,m}^{-2}≜D_{m}\frac{W_{0}}{W(t,\tau )-{\bar{\lambda }}^{2}W(t/T,\tau /T).}$$

Using Equations (26)–(34), figure out the corresponding X, Y and Z, and substitute them into Equation (25) to obtain $V_{AB}$; in this way, we can eventually obtain the value of $K_{ANGO}$.

**Figure 6:**
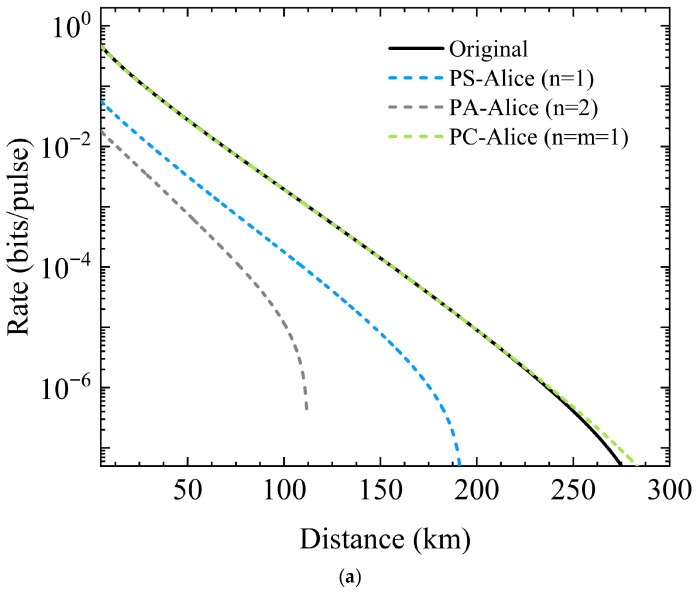

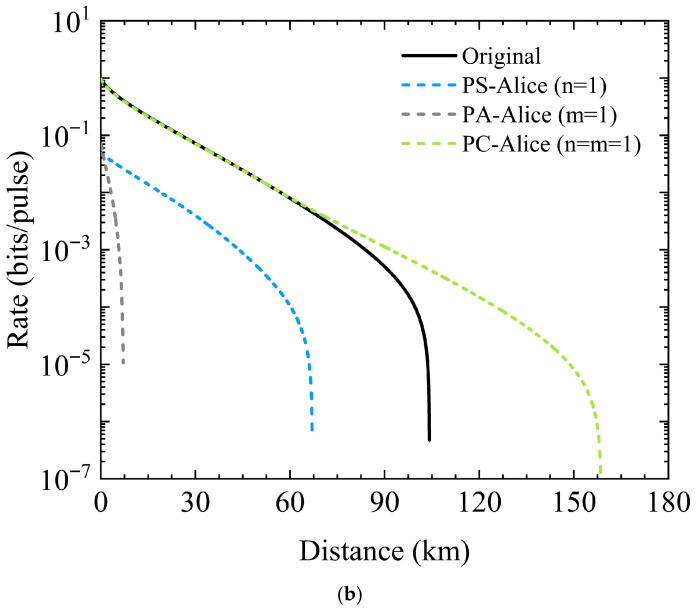
The variation in the SKR with the transmission distance when three non-Gaussian operations are implemented at Alice’s side: (**a**) $\eta_{0}=0.01$; (**b**) $\eta_{0}=0.02$.

The variation in the SKR with the transmission distance when three non-Gaussian operations are implemented at Alice’s side is shown in Figure 6. Here, $\eta_{0}$ is the transmissivity of the attenuator. In Figure 6a and Figure 6b, $\eta_{0}=0.01$ and $\eta_{0}=0.02$ respectively, the fiber loss is 0.2 dB/km, the reconciliation efficiency β=0.95, and the excess noise ξ=0.01. The blue, gray, and green dashed lines represent photon subtraction (PS), photon addition (PA), and photon catalysis (PC), respectively. For comparison, traditional PSP-CVQKD is also shown by the black solid line.

As shown in Figure 6a, the key performance of single-photon catalysis (n=m=1) has high linearity and far exceeds that of the other two non-Gaussian operations. It is almost the same as the performance of PSP-CVQKD without non-Gaussian operations, but has a slight advantage in the maximum transmission distance. When n=1, the performance of photon subtraction is between that of photon catalysis and photon addition. When n=2, the performance of photon addition is inferior to photon subtraction and far worse than photon catalysis. This is also applicable in Figure 6b. The fundamental reason is that, compared with photon catalysis, both photon addition and photon subtraction introduce more noise, especially photon addition, thus resulting in the poorest key performance.

It can be observed in Figure 6b that the increase in $\eta_{0}$ leads to a significant reduction in the transmission distance, with each curve showing a distinct and steep inflection point. However, starting from around 100 km of transmission distance, the SKR of single-photon catalysis is significantly better than that of PSP-CVQKD without non-Gaussian operations, and is even far ahead of photon subtraction and photon addition. The performance of photon addition is the poorest, with a transmission distance of less than 10 km, almost unusable. In summary, among the three non-Gaussian operations, photon addition not only fails to improve the system performance but also greatly reduces the transmission distance. The photon subtraction performance lies between photon addition and photon catalysis, and its maximum transmission distance is also shorter than the original scheme. However, when the compression parameter is large, it can improve the overall performance. Under the condition that the compression parameter and excess noise remain unchanged, the photon catalysis operation performs most prominently in increasing the transmission distance and enhancing the key rate.

## 6. PSP-CVQKD Scheme Based on Non-Gaussian Operations Implemented at Bob’s Side

**Figure 7:**
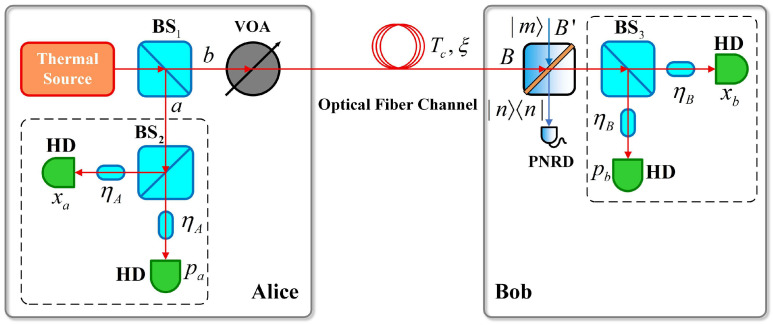
PSP-CVQKD scheme based on non-Gaussian operations implemented at Bob’s side.

Figure 7 shows the scheme of implementing non-Gaussian operations at the Bob’s side. 

It is interesting that the method presented in Section 5 is not only applicable to the scenario where non-Gaussian operations are performed at Alice’s side (with $T_{c}=1$ and ξ=0), but also to those at Bob’s side, and even to the traditional PSP-CVQKD protocol without non-Gaussian operations (with T=1 and m=n).

Using the method in Section 5, Equation (4) can be rewritten as

(35) $$\chi_{out}(\alpha ,\beta )=\exp[(-b_{1}|\alpha {|}^{2}+b_{2}|\beta {|}^{2})/2+b_{3}(\alpha \beta +\alpha^{*}\beta^{*})/2],$$

Then, taking $\chi_{out}(\alpha ,\beta )$ as the input of BS, the output of BS can be obtained as

(36) $$\chi_{BS-out}(\alpha ,\beta )=\chi_{out}(\alpha ,\beta )G_{m,n}(\alpha ,\beta ),$$

The normalized CF can be written as

(37) $$\chi {'}_{BS-out}(\alpha ,\beta )=\frac{\chi_{out}(\alpha ,\beta )G_{m,n}(\alpha ,\beta )}{G_{m,n}(0,0)}.$$

By setting n=0, m=0, and m=n respectively, we can obtain the success probabilities for photon addition, photon subtraction, and photon catalysis respectively:

(38) $$G_{m,0}(0,0)=\frac{2{\left(b_{2}+1\right)}^{m}R^{m}}{{\left(1+T+Rb_{2}\right)}^{m+1}},$$

(39) $$G_{0,n}(0,0)=\frac{2{\left(b_{2}-1\right)}^{n}R^{n}}{{\left(1+T+Rb_{2}\right)}^{n+1}},$$

(40) $$G_{m,m}(0,0)=\frac{2{\left(1+T-Rb_{2}\right)}^{m}}{{\left(1+T+Rb_{2}\right)}^{m+1}}P_{m}(\frac{D_{3}^{2}+1}{D_{3}^{2}-1}).$$

$P_{m}(\cdot )$ is the mth Legendre polynomial. When single-photon catalysis (m=n=1) is implemented, Equation (40) can be further simplified to

(41) $$G_{1,1}(0,0)=\frac{8T+2R^{2}(b_{2}^{2}-1)}{{\left(1+T+Rb_{2}\right)}^{3}}.$$

It is easy to see that when $T_{c}=1$ and ξ=0, since the optical fiber channel poses no obstruction to the optical signal and there is no noise, it is equivalent to extending Alice’s boundary to the right side of Bob’s BS. At this point, the non-Gaussian operations at Bob’s side are equivalent to being performed at Alice’s side.

**Figure 8:**
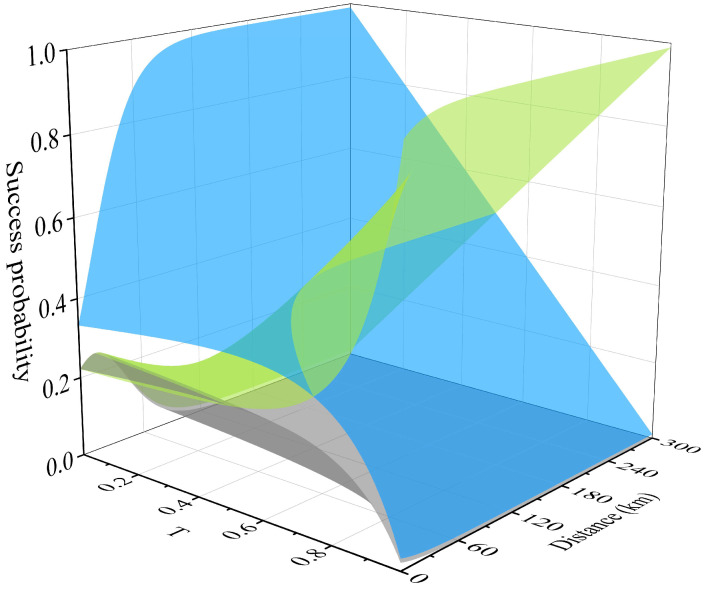
The success probabilities of Bob’s implementation of three non-Gaussian operations vary with the transmission distance and transmissivity; the gray surface, blue surface and green surface represent the success probability of the photon addition, photon subtraction and photon catalysis, respectively.

To demonstrate the factors influencing the success probability of non-Gaussian operations and the extent of their impact more intuitively, we utilized Equations (38)–(40) to plot the relationship between the success probability and the transmissivity T as well as the transmission distance L, as shown in Figure 8. It can be seen that regardless of how T is adjusted, the success probability of the photon addition is the lowest, with the maximum success probability being only about 25%, and it rapidly decreases with the increase in L, reaching almost zero at approximately 90 km. In the small transmissivity interval, the success probability of the photon subtraction is very high, approximately 98%. As T increases, the success probability almost linearly decreases, and it reduces to zero when T approaches 100%. When T is small and fixed, the success probability increases with the increase in L and then remains constant. The situation is more complex for photon catalysis. When the transmission distance is very short, the success probability slightly decreases with the increase in transmissivity and then rapidly increases, while in the range where L exceeds 120 km, the success probability basically increases linearly with the increase in transmissivity. Moreover, when T approaches 100%, the change in L no longer affects the success probability, which remains at approximately 98%. It should be noted that in the experiments corresponding to Figure 5 and Figure 8, we assume that PNRD is ideal, that is, the detection efficiency is 100%. However, in practical applications, due to dark counts and limited quantum efficiency, the actual efficiency of PNRD is often within the range of 90% to 95%. Obviously, a lower detection efficiency will result in a lower effective SKR. In this experiment, an extra BS can be added before the PNRD (see Figure 3); by changing the transmissivity of the BS, the detection efficiency of the PNRD can be modified.

The different success probabilities will affect the system SKR when non-Gaussian operations are implemented at Bob’s side:

(42) $$K_{BNGO}=P_{NGO}(\beta_{RECO}I_{AB}-\chi_{BE}).$$

By using the same method as in Section 5, the CM expressions are derived as

(43) $$V_{AB-a}=\left(\begin{array}{cc} \left[2m\frac{Tb_{3}^{2}}{\left(1+T+Rb_{2})(b_{2}+1\right)}+\tau_{1}\right]I & (m+1)\tau_{3}\sigma_{Z}\\ (m+1)\tau_{3}\sigma_{Z} & \left[2m\frac{b_{2}+1}{1+T+Rb_{2}}+\tau_{2}\right]I \end{array}\right),$$

(44) $$V_{AB-s}=\left(\begin{array}{cc} \left[2n\frac{b_{3}^{2}}{\left(1+T+Rb_{2})(b_{2}-1\right)}+\tau_{1}\right]I & (n+1)\tau_{3}\sigma_{Z}\\ (n+1)\tau_{3}\sigma_{Z} & \left[2n\frac{T}{\left(1+T+Rb_{2})(b_{2}-1\right)}+\tau_{2}\right]I \end{array}\right),$$

and

(45) $$V_{AB-c}=\left(\begin{array}{cc} (\frac{{\hat{D}}_{m,m}E_{t}C_{1}^{2}}{{\hat{D}}_{m,m}E_{t}}+\tau_{1})I & (\frac{{\hat{D}}_{m,m}E_{t}C_{1}C_{2}}{{\hat{D}}_{m,m}E_{t}}+\tau_{3})\sigma_{Z}\\ (\frac{{\hat{D}}_{m,m}E_{t}C_{1}C_{2}}{{\hat{D}}_{m,m}E_{t}}+\tau_{3})\sigma_{Z} & (\frac{{\hat{D}}_{m,m}E_{t}C_{2}^{2}}{{\hat{D}}_{m,m}E_{t}}+\tau_{2})I \end{array}\right).$$

**Figure 9:**
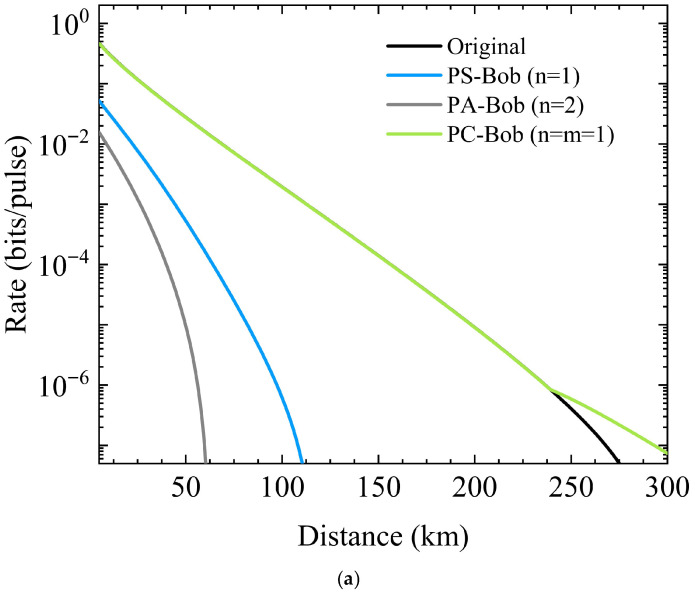

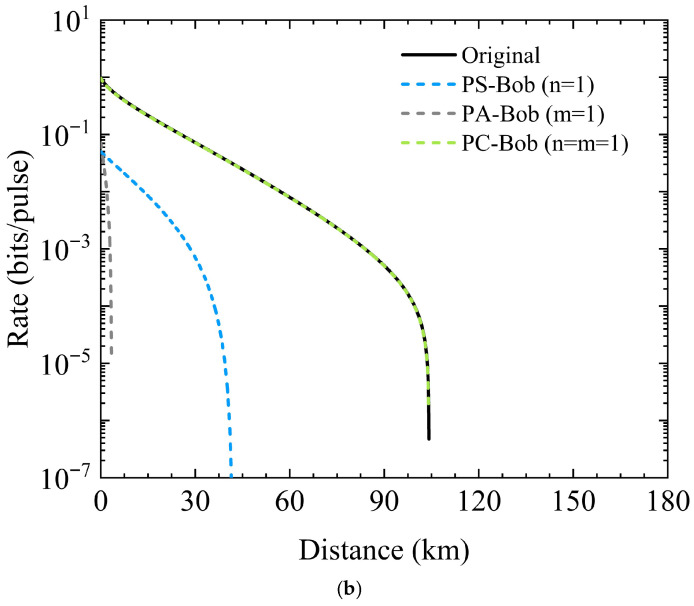
The variation in the SKR with the transmission distance when three non-Gaussian operations are implemented at Bob’s side: (**a**) $\eta_{0}=0.01$; (**b**) $\eta_{0}=0.02$.

Using the CM, we calculate SKR and present its variation with the transmission distance in Figure 9. The parameters such as $\eta_{0}$, fiber loss, β, and ξ are the same as those in Figure 6.

The blue, gray, and green solid lines represent photon subtraction (PS), photon addition (PA), and photon catalysis (PC) respectively. The traditional PSP-CVQKD represented by black solid line is also shown. As shown in Figure 9a, the key performance of single-photon catalysis (n=m=1) is far superior to that of photon subtraction and photon addition. Combined with Figure 6a, it can be seen that when the transmission distance is less than 240 km, the performance of single-photon catalysis implemented at either Alice’s or Bob’s side is the same as that of PSP-CVQKD without non-Gaussian operations. Beyond 240 km, the performance of single-photon catalysis implemented at Bob’s side is the best, followed by that at Alice’s side, and the traditional PSP-CVQKD is slightly inferior. When n=1, the performance of photon subtraction is far behind that of single-photon catalysis. When n=2, the key performance of photon addition is the worst. Interestingly, the key performance of the corresponding operations implemented at Bob’s side is much worse than that at Alice’s side. This is because more noise is introduced after channel transmission, which affects the performance of non-Gaussian operations. In contrast, the photon catalysis operation implemented at the receiving end shows better performance than those at the sending end. This is because the catalysis operation is equivalent to a noiseless linear amplifier, which actually improves the purity of the quantum state and thus enhances the key performance.

As the VOA transmissivity reaches 0.02, the key performance of the three non-Gaussian operations drops significantly, as shown in Figure 9b. Unlike at Alice’s side, the key performance of photon-catalysis at Bob’s side, although leading the other two non-Gaussian operations, remains the same as the original scheme, neither improving nor deteriorating. The performance of the photon addition is the poorest and completely unusable, while photon subtraction lies between the first two operations.

## 7. Conclusions

This paper proposed an improved PSP-CVQKD scheme based on non-Gaussian operations to enhance the transmission performance and key performance of the system. We introduced the success probabilities of non-Gaussian operations, as well as the input–output relationship in the original PSP-CVQKD scheme, based on which we derived the input–output relationship, CM, and SKR calculation method for the PSP-CVQKD scheme with non-Gaussian operations implemented at the transmitter and receiver respectively. We also investigated and simulated the factors affecting the success probability of non-Gaussian operations. Finally, we simulated and compared the key performances, and studied the factors affecting them. In conclusion, when implementing non-Gaussian operations at the sender, we found that implementing single-photon catalysis and appropriately increasing the transmissivity of the BS can significantly improve the success probability, which is very beneficial for enhancing the SKR and increasing the transmission distance. We also found that a smaller VOA transmissivity can achieve a higher SKR or a longer transmission distance. When the transmissivity of the VOA remains unchanged, photon catalysis can achieve the highest SKR and the maximum transmission distance, whether the non-Gaussian operation is implemented at Alice’s or Bob’s side. If photon catalysis cannot be implemented, then implementing photon subtraction at the sender’s side is a wise choice. Photon addition is almost useless. The reason why the performance of implementing photon subtraction or photon addition is far inferior to that of photon catalysis is that the former two introduce a lot of noise, which seriously affects the system performance.

The analysis method proposed in this paper is feasible and has practical reference value in improving the key performance of the system. Our scheme provides a new idea for further in-depth research on the performance improvement of non-Gaussian operations and PSP-CVQKD.

## Data Availability

All data generated or analyzed during this study are included in this published article.
